# A propensity score matching analysis for cardio metabolic risk of antipsychotics in patients with schizophrenia using Japanese claims data

**DOI:** 10.1186/s12888-020-02987-1

**Published:** 2020-12-09

**Authors:** Ichiro Kusumi, Sachie Inoue, Kenji Baba, Tadashi Nosaka, Toshihisa Anzai

**Affiliations:** 1 Department of Psychiatry, Hokkaido University Graduate School of Medicine, Kita 15, Nishi 7, Kita-ku, Sapporo, Hokkaido Japan; 2 CRECON Medical Assessment Inc, 2-12-15, Shibuya, Shibuya-ku, Tokyo, Japan; 3 Sumitomo Dainippon Pharma Co., Ltd., 13-1, Kyobashi 1-Chome, Chuo-ku, Tokyo, Japan; 4 Department of Cardiovascular Medicine, Hokkaido University Graduate School of Medicine, Kita 15, Nishi 7, Kita-ku, Sapporo, Hokkaido Japan

**Keywords:** Schizophrenia, Atypical antipsychotic drugs, Cardio-metabolic risk, Monitoring, Claim database, Propensity score matching

## Abstract

**Background:**

The aim of this study was to evaluate the cardio-metabolic risk in schizophrenia patients treated by atypical antipsychotic drugs compared with that in those treated without atypical antipsychotic drugs using a nationwide insurance claims database and medical examination database in Japan.

**Methods:**

Eligible patients were defined as those meeting the following two criteria: (i) A diagnosis of schizophrenia (ICD-10 code: F20) was made between 1 January 2005 and 31 December 2017, with data available for at least 6 months before the diagnosis was made (index month), and (ii) health check-up data were available within ±3 months of the index month. The primary endpoint was changes in cardio-metabolic risk based on the Suita score at 1 year, and the secondary endpoints were changes in medical examination data related to cardio-metabolic risk (total cholesterol [TC], triglyceride, high-density lipoprotein cholesterol, low-density lipoprotein cholesterol, body mass index [BMI], and hemoglobin A1c) at 1 year. The primary endpoint was evaluated by multivariate analysis, with the cumulative chlorpromazine equivalent amount and the baseline Suita score added as covariates.

**Results:**

One-hundred eighty five pairs of propensity score (PS)-matched patients were evaluated. Patients receiving atypical antipsychotic drugs exhibited a greater change in the Suita score and a risk of coronary heart disease based on the Suita score of 0.530 and 0.098%, respectively, than patients not receiving atypical antipsychotic drugs, but there was no significant difference (*p* = 0.412 and 0.610). The significant changes in TC and BMI were determined as 6.525 mg/dL and 0.380 kg/m^2^ greater, respectively, in patients treated with atypical antipsychotic drugs (*p* = 0.037 and 0.011).

**Conclusions:**

There were no significant increases in changes in the Suita score at 1 year by treatment with atypical antipsychotic drugs compared with treatment without atypical antipsychotic drugs. However, the TC and BMI were significantly higher in patients treated with atypical antipsychotic drugs.

**Supplementary Information:**

The online version contains supplementary material available at 10.1186/s12888-020-02987-1.

## Background

Schizophrenia is a chronic and severe mental disorder affecting 20 million people worldwide [1]. In Japan, the estimated number of patients currently receiving treatment is 795,000 and the lifetime morbidity rate is 0.7% [2]. Schizophrenia is associated with a broad array of symptoms such as hallucinations and delusions (positive symptoms), abulia and autism (negative symptoms), cognitive impairment, and affective disorder (depressive symptoms). It is associated with considerable disability, which affects educational and occupational performance. Schizophrenia is commonly seen in persons in their late teens to 30s.

Antipsychotic drugs used in the treatment of schizophrenia are classified into two types: first-generation (typical) antipsychotic drugs, which have conventionally been used, and second-generation (atypical) antipsychotic drugs. All antipsychotic drugs block dopamine D2 receptors. Put simply, however, typical antipsychotic drugs block dopamine D2 receptors alone, whereas atypical antipsychotic drugs also act on serotonin 5-HT2A receptors and other neurotransmitters [3]. Atypical antipsychotic drugs have fewer extrapyramidal side-effects than typical antipsychotic drugs and are used as first-line antipsychotic treatment [4, 5]. However, weight gain and impaired glucose tolerance (IGT) have developed as adverse reactions to atypical antipsychotic drugs, and there are cases in which medication is discontinued due to the onset of diabetes, ketoacidosis, or a decrease in quality of life (QOL) [6]. The blockage of receptors, such as histamine H1, serotonin 5-HT2A and 5-HT2C, muscarinic M3, and adrenergic α1 and α2, is implicated in the onset of weight gain in patients receiving atypical antipsychotic drugs, and involvement of genetic factors has also been pointed out [7].

Leucht et al. performed a meta-analysis of the safety of antipsychotic drugs, including overall efficacy, relapse, QOL, extrapyramidal side-effects, weight gain, and sedation, in patients with different psychiatric symptoms based on 150 placebo-controlled randomized controlled trials, and reported that the standardized mean difference (SMD) in weight gain was greater with atypical antipsychotic drugs than with typical antipsychotic drugs [8]. Other studies have also reported that treatment with atypical antipsychotics in patients with schizophrenia lead to abnormal changes in metabolic parameter such as waist circumference, body mass index, and lipid or glycemic index as well as body weight, even for a short term of their use [9, 10]. In common, these studies explain the importance of initiating and periodically continuing of monitoring when using atypical antipsychotics to in those patients in order to minimize the risk of metabolic syndrome development. Although there is evidence suggesting the effects of antipsychotic drugs on body weight gain and metabolic syndrome as described above, there is still little suggesting the relationship between atypical antipsychotic drugs and cardio-metabolic risk such as myocardial infarction or new-onset diabetes.

In this study, we hypothesized that cardio-metabolic risk is higher in schizophrenia patients treated with atypical antipsychotic drugs than in those treated without atypical antipsychotic drugs, and retrospectively examined this hypothesis using health insurance claims data and medical examination data.

## Methods

### Study design

This retrospective cohort study utilized a nationwide insurance claims database and medical examination database in Japan constructed by JMDC Inc. [11] The JMDC database is an epidemiological receipt database that has accumulated receipts (inpatient, outpatient, and dispensing) and medical examination data received from multiple employer-based health insurance associations since 2005. The cumulative dataset is approximately 5.6 million subjects as of June 2018. The database can track data for each patient in chronological order, even if the patient visited or was hospitalized at multiple medical institutions. As the database is composed of employer-based health insurance data, it contains insurance claims data of the employers and their families, and is biased in terms of age distribution, which is a point of concern. However, approximately 80% of the patients with schizophrenia in this study were younger than 65 years [12], and it was therefore reasonable to use the JMDC database for this study.

The ethics review committee secretariat of Hokkaido University stated that no ethics review was necessary for this study because it was a secondary analysis of an anonymous patient database.

### Study population

From the JMDC claims database of approximately 59,994 patients with schizophrenia, schizotypal, delusional, and other non-mood psychotic disorders (ICD-10 code: F20–29) between January 1st, 2005 and December 31st, 2017, we identified patients who were newly diagnosed with schizophrenia and for whom medical examination data were available during the period. Eligible patients were defined by meeting the following two criteria; (i) A diagnosis of schizophrenia (ICD-10 code: F20) made between January 1st, 2005 and December 31st, 2017, with data available for at least 6 months before the diagnosis was made (index month), and (ii) medical examination data were available within ±3 months of the index month. Patients meeting the following criteria were excluded from the study: (i) Patients with a suspected diagnosis of schizophrenia (ICD-10 code: F20); (ii) patients for whom an antipsychotic drug (ATC code: N05A) was prescribed within 6 months before the index month; (iii) patients who were 65 years or older at the index month; or (iv) patients for whom an anti-dementia drug (ATC code: N06D.X) was prescribed within 6 months before the index month. The exclusion criteria were defined for the following reasons: To identify patients receiving an antipsychotic drug for newly diagnosed schizophrenia [(i) and (ii)]; to exclude patients for whom an antipsychotic drug was used for delirium [(iii)]; and to exclude patients for whom an antipsychotic drug was used for dementia [(iv)]. The analysis population and treatment groups were defined by each of the study outcome.

### Study outcome and analysis population

#### Primary endpoint

The primary endpoint was changes in cardio-metabolic risk based on the Suita score at 1 year, and changes in the Suita score from baseline to 1 year were compared between groups treated with and without atypical antipsychotic drugs. The baseline was defined as the first month in which an atypical antipsychotic drug was prescribed for the group treated with atypical antipsychotic drugs, or the index month for the group treated without atypical antipsychotic drugs. The Suita score, developed by a research team at the National Cerebral and Cardiovascular Center, is a risk score to predict the 10-year risk for the development of coronary heart disease (CHD), such as myocardial infarction, in Japanese persons, and it is calculated based on age, sex, smoking habits, diabetes status, blood pressure, low-density lipoprotein cholesterol (LDL-C), high-density lipoprotein cholesterol (HDL-C), and estimated glomerular filtration rate (eGFR) [13]. The Suita score was used to calculate the risk of developing CHD, and changes in the risk of developing CHD from baseline to 1 year were also evaluated.

Among the eligible patients, those for whom medical examination data were available within ±3 months of 1 year after the index month and the calculation of the Suita score was possible (medical examination follow-up population) were subjected to evaluation of the primary endpoint. The group treated with atypical antipsychotic drugs was defined as patients for whom an atypical antipsychotic drug was prescribed in the index month or the following month and the medication was not switched to a typical antipsychotic drug. The group treated without atypical antipsychotic drugs was defined as patients for whom no atypical antipsychotic drug was prescribed within 12 months from baseline.

#### Secondary endpoint

The secondary endpoints were changes in medical examination data related to cardio-metabolic risk from baseline until 1 year, changes in the Framingham and Japan Arteriosclerosis Longitudinal Study (JALS) scores from baseline until 1 year, and the risk of developing diabetes, cardiovascular events, and cardiovascular death during the observation period. The medical examination data for cardio-metabolic risk were total cholesterol (TC), triglyceride (TG), HDL-C, LDL-C, body mass index (BMI), and hemoglobin A1c (HbA1c), and they were evaluated using medical examination data at baseline and 1 year. The Framingham score and JALS score are risk scores to predict the risk for the development of CHD [14, 15], and the scores were calculated using medical examination data. The onset of diabetes was defined as the diagnosis of diabetes (ICD-10 code: E10–14) and the prescription of anti-diabetes drugs (ATC code: A10) after baseline. Cardiovascular events included myocardial infarction, stroke, and hospitalization for heart failure, and patients were considered to have a cardiovascular event if they had myocardial infarction (ICD-10 code: I21) or underwent surgery for myocardial infarction, if they were diagnosed with stroke (ICD-10 code: I61.4, I61.9, I63.3–I63.9, and I64), or if they were hospitalized for heart failure (ICD-10 code: I11.0, I50.0, I50.1, and I50.9) after baseline. Cardiovascular deaths were defined as deaths among those with cardiovascular events.

Biomarkers, and Framingham and JALS scores were evaluated in the medical examination follow-up population. Onset of diabetes, cardiovascular events and cardiovascular death were analyzed in all eligible patients (long-term follow-up population). In the long-term follow-up population, the group treated with atypical antipsychotic drugs was defined as patients for whom an atypical antipsychotic drug was prescribed in the index month or the following month (excluding as-needed usage), and the group treated without atypical antipsychotic drugs was defined as patients for whom an atypical antipsychotic drug was not prescribed in the index month or the following month.

#### Clinical monitoring

The frequency of monitoring diabetes and lipid markers was also evaluated to determine whether physicians treating schizophrenia monitored diabetes and dyslipidemia regularly. Diabetes monitoring was defined as follows: “Urinary qualitative/semi-quantitative examination (general substances),” “HbA1c measurement,” or “measurement of glycated albumin or 1,5-anhydro-D-glucitol” was performed at the hospital where schizophrenia was diagnosed or at the medical institution where an antipsychotic drug was administered. Lipid marker monitoring was defined as follows: Biochemical examinations of blood (medical remuneration code: D007) conducted at the institution where schizophrenia was diagnosed or atypical antipsychotic was prescribed.

### Statistical analysis

To reduce the effects of potential confounding factors in this observational study, a propensity score (PS) matching analysis was applied in both the medical examination follow-up population and the long-term follow-up population. PS matching reduces bias due to confounding factors by matching patients on baseline variables using a multivariable logistic regression model. Confounding factors of patient characteristics were examined based on clinical findings, theoretical grounds, and findings from previous studies [16, 17]. The candidate factors examined are shown in the Additional file. Candidate factors were narrowed down by the following procedure: (1) Diseases that had affected a higher proportion of patients or drugs with a high prescription rate (2% or higher); (2) presence of biases between the two groups (*p* < 0.25 for between-group difference); and (3) among variables satisfying (1) and (2) above, those clinically related to each other (eg, “antidepressant use” and “depression”) were narrowed down to a single variable. The following factors were selected: sex, year of diagnosis, history of diabetes, age at baseline, systolic blood pressure, LDL-C, prescription of corticosteroids or antidepressants during the 1 month before baseline, hyperthyroidism, chronic hepatitis, myocardial infarction or other CHD, heart failure, peripheral vascular disease, and cancer other than risk factors during the 6 months before baseline. The PS matching pairs were created at a ratio of 1:1 based on the nearest neighbor matching algorithm with a 0.25-caliper distance with no replacements. The goodness-of-fit of the logistic regression model was evaluated by the Hosmer-Lemeshow test. Discrimination, ie, the capability to classify individuals with and without events, was evaluated by the C-statistic or the area under the receiving operating characteristic curve [18]. A C-statistic > 0.7 indicates good discrimination.

Regarding the primary endpoint, changes in the Suita score from baseline to 1 year and the difference in event incidence risk calculated from the score are presented as the mean ± standard deviation (SD). The relationship was evaluated by multivariate analysis using changes in the Suita score from baseline to 1 year and the difference in event incidence risk calculated from the score as the objective variables, and the presence of a new prescription of an atypical antipsychotic drug as an explanatory variable, with the cumulative chlorpromazine equivalent amount and the baseline Suita score added as covariates.

Changes in several medical examination data at 1 year as a secondary endpoint are also presented as the mean ± SD. In addition, multivariate analysis was performed using changes in each medical examination data at 1 year as the objective variable and the presence of a new prescription of an atypical antipsychotic drug as an explanatory variable, with the cumulative chlorpromazine equivalent amount and medical examination data values at baseline added as covariates. Cox’s proportional hazards model was used to calculate the hazard ratios for the onset of diabetes, cardiovascular events, and cardiovascular death during the observation period in the group treated with atypical antipsychotic drugs compared with that treated without atypical antipsychotic drugs, with the presence of a new prescription of an atypical antipsychotic drug as an explanatory variable. The cumulative chlorpromazine equivalent was adjusted as a covariate.

In the evaluation of the frequency of monitoring diabetes and lipid markers, between-group comparisons were performed using the Student’s t-test as appropriate. All data analyses were carried out with SAS v.9.4, and *P*-values of < 0.05 were considered significant.

## Results

### Study population and analysis population

**Fig. 1:**
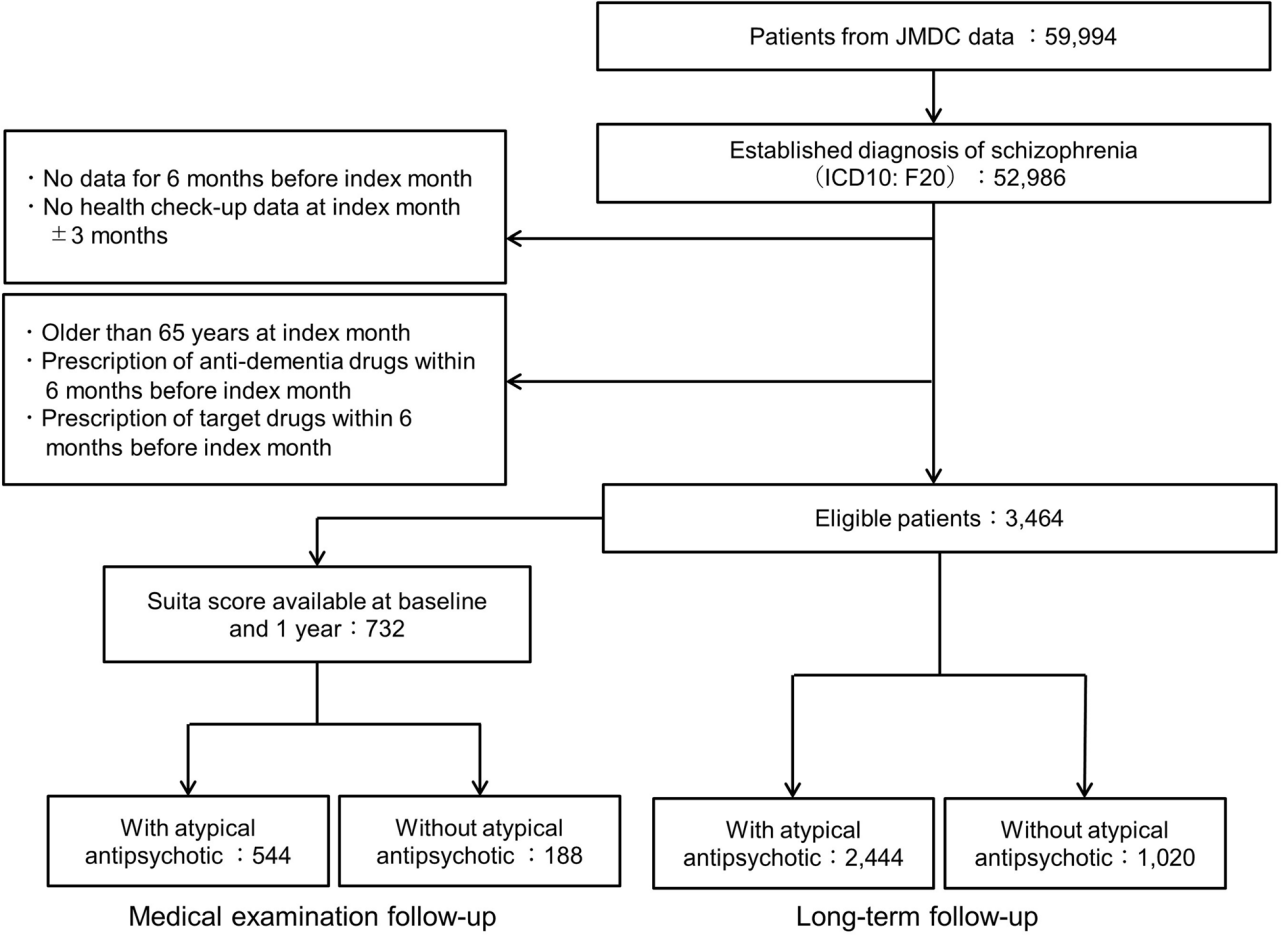
Patients flowchart

A flow chart of the study population is shown in Fig. 1. In the JMDC database, 52,986 patients having schizophrenia (F20) were identified during the study period. Of them, 3464 patients who were newly diagnosed with schizophrenia and having medical examination data were identified as eligible patients. The numbers of patients treated with and without atypical antipsychotic drugs at baseline were 2444 and 1020, respectively (long-term follow-up population). Of these, the number of patients who had baseline and 1-year Suita scores (i.e., medical examination follow-up population) was 732. The number of patients evaluated for the primary endpoint was 544 and 188 in the group treated with and without atypical antipsychotic drugs, respectively (Fig. 1).

Among the eligible patients (*n* = 3464), logistic regression analysis was performed for PS matching. SMDs of some covariates between with and without atypical antipsychotic groups in PS matched population were over 0.1. However, since the distribution of propensity score of the two groups were well overlapped, it was evaluated that the covariates were balanced after PS matching based on a comprehensive judgement. The model was well-calibrated (Hosmer-Lemeshow test = 0.512) and demonstrated good discrimination (C-statistic = 0.770). The characteristics of PS-matched medical examination follow-up patients (*n* = 370, 185 pairs of patients), and PS-matched long-term follow-up patients (*n* = 1778, 889 pairs of patients) were summarized in Table 1. The mean age of the PS-matched medical examination follow-up patients was 47 years, and male patients accounted for approximately 70%. Laboratory values were generally within the normal range. The proportions of patients with a history of diabetes and smoking habits were 4.9 and 27.8%, respectively.

**Table 1:** Patient characteristics

	Eligible patients		Medical examination follow-up patients (PS matched)		Long-term follow-up patients (PS matched)	
	With atypical antipsychotic (*N* = 2444)	Without atypical antipsychotic (*N* = 1020)	With atypical antipsychotic (*N* = 185)	Without atypical antipsychotic (*N* = 185)	With atypical antipsychotic (*N* = 889)	Without atypical antipsychotic (*N* = 889)
Age						
< 25 years	131 (5.4%)	51 (5.0%)	0	0	14 (1.6%)	26 (2.9%)
25–29 years	244 (10.0%)	67 (6.6%)	0	0	47 (5.3%)	54 (6.1%)
30–34 years	280 (11.5%)	88 (8.6%)	0	0	67 (7.5%)	76 (8.6%)
35–39 years	353 (14.4%)	108 (10.6%)	38 (20.5%)	28 (15.1%)	112 (12.6%)	99 (11.1%)
40–49 years	947 (38.8%)	369 (36.2%)	85 (46%)	91 (49.2%)	381 (42.9%)	345 (38.8%)
50–59 years	443 (18.1%)	279 (27.4%)	55 (29.7%)	59 (31.9%)	235 (26.4%)	245 (27.6%)
> =60 years	46 (1.9%)	58 (5.7%)	7 (3.8%)	7 (3.8%)	33 (3.7%)	44 (5%)
Mean (years)	40.9 ± 9.8	43.9 ± 10.5	46.3 ± 7	47.0 ± 6.8	44.4 ± 9.1	44.3 ± 9.8
Sex (male %)	1608 (65.8%)	676 (66.3%)	127 (68.6%)	133 (71.9%)	594 (66.8%)	570 (64.1%)
Year of diagnosis						
-2007	25 (1.0%)	15 (1.5%)	0	0	6 (0.7%)	6 (0.7%)
2008–2010	327 (13.4%)	83 (8.1%)	16 (8.7%)	21 (11.4%)	69 (7.8%)	70 (7.9%)
2011–2013	693 (28.4%)	249 (24.4%)	62 (33.5%)	47 (25.4%)	229 (25.8%)	226 (25.4%)
2014-	1399 (57.2%)	673 (66%)	107 (57.8%)	117 (63.2%)	585 (65.8%)	587 (66%)
SBP (mmHg)	118.2 ± 15.4	119.6 ± 16.3	120.5 ± 14.9	121.1 ± 16.4	119.5 ± 16.3	119.1 ± 16
DBP (mmHg)	72.9 ± 11.4	73.9 ± 11.5	75.1 ± 10.9	75.8 ± 12.2	74.3 ± 11.7	73.9 ± 11.4
LDL-C (mg/dL)	119.6 ± 32.5	118.4 ± 32.5	118.6 ± 29.4	123 ± 32.1	118.4 ± 31.6	118.7 ± 32.1
HDL-C (mg/dL)	62.8 ± 17.5	62.3 ± 17.0	63.3 ± 16.4	62.2 ± 15.8	62.6 ± 18	62.7 ± 16.8
eGFR (mL/min/1.73 m^2^)	98.3 ± 18.1	96.7 ± 19.5	92.7 ± 16.4	94.7 ± 18.3	96.7 ± 17.7	97.5 ± 19
HbA1c (%)	5.5 ± 0.6	5.5 ± 0.8	5.6 ± 0.7	5.5 ± 0.6	5.5 ± 0.7	5.5 ± 0.7
TC (mg/dL)	205.5 ± 39.0	203.1 ± 38.4	205.2 ± 33.9	207.6 ± 35.9	204 ± 38.5	203.4 ± 37.3
TG (mg/dL)	120.5 ± 106.9	118 ± 100.3	126.8 ± 131	123.8 ± 85.9	119.8 ± 105.5	115.4 ± 86.9
BMI (kg/m^2^)	22.8 ± 3.9	22.9 ± 4.0	23.4 ± 3.9	23.4 ± 4.3	23 ± 4.1	22.9 ± 4
Suita score (LDL)	30.5 ± 10.4	32.8 ± 11.0	31.4 ± 10.4	32.7 ± 10.3	31.8 ± 10.4	32.2 ± 10.9
Framingham score (LDL)	0.2 ± 5.0	1.2 ± 5.0	1.1 ± 4.3	1.7 ± 4.1	1.0 ± 4.8	1.0 ± 5.1
JALS score	20.3 ± 15.3	22.6 ± 16.1	21.1 ± 14.7	22.1 ± 14.9	21.2 ± 14.9	21.8 ± 15.9
Smoking habits	646 (26.4%)	274 (26.9%)	51 (27.6%)	52 (28.1%)	245 (27.6%)	246 (27.7%)
Diabetes	88 (3.6%)	60 (5.9%)	13 (7%)	5 (2.7%)	46 (5.2%)	44 (4.9%)

*PS* Propensity score, *SBP* Systolic blood pressure, *DBP* Diastolic blood pressure, *LDL-C* Low-density lipoprotein cholesterol, *HDL-C* High-density lipoprotein cholesterol, *eGFR* Estimated glomerular filtration rate, *HbA1c* Hemoglobin A1c, *TC* Total cholesterol, *TG* Triglyceride, *BMI* Body mass index, *JALS* Japan Arteriosclerosis Longitudinal Study

### Primary endpoint

The changes in the Suita score at 1 year were 1.4 ± 6.3 and 0.7 ± 5.5 in the group treated with and without atypical antipsychotic drugs, respectively (mean ± SD). On multivariate analysis, the changes in the Suita score and the estimated CHD risk based on the Suita score were 0.530 and 0.098% greater, respectively, in the group treated with atypical antipsychotic drugs, but there was no significant difference (*p* = 0.412 and 0.610, adjusted for cumulative chlorpromazine equivalent amount and the baseline Suita score) (Tables 2 and 3).

**Table 2:** Change in Suita score for 1 year. Mean change from baseline

	With atypical antipsychotic			Without atypical antipsychotic		
	N	Mean	SD	N	Mean	SD
Change in Suita score (LDL)	185	1.416	6.322	185	0.681	5.494

*LDL *Low-density lipoprotein, *SD* Standard deviation

**Table 3:** Change in Suita score for 1 year. Results of multivariate analysis (*N* = 370)

Variable	Estimate	95%CI	*P*-value
Change in Suita score (LDL)			
With atypical antipsychotic	0.530	(−0.738, 1.798)	0.412
Cumulative chlorpromazine equivalent	−0.003	(− 0.221, 0.216)	0.980
Baseline Suita score (LDL)	−0.167	(− 0.224, − 0.111)	<.0001
Change in CHD risk by Suita score (LDL) (%)			
With atypical antipsychotic	0.098	(−0.280, 0.477)	0.610
Cumulative chlorpromazine equivalent	0.018	(−0.047, 0.083)	0.589
Baseline Suita score (LDL)	−0.030	(−0.047, − 0.013)	0.001

*LDL* Low-density lipoprotein, *CI* Confidence interval, *CHD* Coronary heart disease

### Secondary endpoint

The changes in medical examination data (TC, TG, HDL-C, LDL-C, BMI, and HbA1c) at 1 year in the group treated with and without atypical antipsychotic drugs were 7.049 ± 31.308 mg/dL and 1.133 ± 30.177 mg/dL (TC), 5.589 ± 130.910 mg/dL and 5.173 ± 103.008 mg/dL (TG), − 0.568 ± 12.582 mg/dL and − 0.346 ± 10.109 mg/dL (HDL-C), 6.130 ± 25.689 mg/dL and 0.454 ± 25.469 mg/dL (LDL-C), 0.618 ± 1.320 kg/m^2^ and 0.123 ± 1.315 kg/m^2^ (BMI), and − 0.051 ± 0.350% and 0.059 ± 0.036% (HbA1c), respectively (mean ± SD) (Tables 4, 5, 6, 7 and 8). The changes in the Framingham and JALS scores at 1 year were 0.562 ± 2.335 and 0.286 ± 1.967, and 2.433 ± 8.329 and 1.452 ± 7.427, respectively (Table 8).

**Table 4:** Change in TC for 1 year. Mean change from baseline

	With atypical antipsychotic			Without atypical antipsychotic		
	N	Mean	SD	N	Mean	SD
Change in TC (mg/dl)	185	7.049	31.308	185	1.133	30.177

*TC* Total cholesterol, *SD* Standard deviation

**Table 5:** Change in TC for 1 year. Results of multivariate analysis (*N* = 370)

Variable	Estimate	95%CI		*P*-value
With atypical antipsychotic	6.525	(0.406,	12.643)	0.037
Cumulative chlorpromazine equivalent	−0.686	(−1.736,	0.363)	0.199
Baseline TC	−0.399	(−0.480,	− 0.319)	<.0001

*TC* Total cholesterol, *CI* Confidence interval

**Table 6:** Change in BMI for 1 year. Mean change from baseline

	With atypical antipsychotic			Without atypical antipsychotic		
	N	Mean	SD	N	Mean	SD
Change in BMI (kg/m^2^)	185	0.618	1.320	185	0.123	1.315

*BMI* Body mass index, *SD* Standard deviation

**Table 7:** Change in BMI for 1 year. Results of multivariate analysis (*N* = 370)

Variable	Estimate	95%CI		*P*-value
With atypical antipsychotic	0.380	(0.090,	0.670)	0.011
Cumulative chlorpromazine equivalent	0.048	(−0.001,	0.098)	0.057
Baseline BMI	−0.047	(− 0.080,	−0.015)	0.004

*BMI* Body mass index, *CI* Confidence interval

**Table 8:** Change in other outcomes for 1 year

Outcome	Group	Change from baseline			Multivariate analysis			
		N	Mean	SD	Estimate	95%CI		*P*-value
TG (mg/dl)	With atypical antipsychotic	185	5.589	130.910	8.669	(−13.141,	30.478)	0.435
	Without atypical antipsychotic	185	5.173	103.008	–	–	–	–
HDL-C (mg/dl)	With atypical antipsychotic	185	−0.568	12.582	0.104	(−2.359,	2.566)	0.934
	Without atypical antipsychotic	185	−0.346	10.109	–	–	–	–
LDL-C (mg/dl)	With atypical antipsychotic	185	6.130	25.689	5.537	(−0.178,	11.253)	0.058
	Without atypical antipsychotic	185	0.454	25.469	–	–	–	–
HbA1c (%)	With atypical antipsychotic	144	−0.051	0.350	−0.075	(− 0.159,	0.008)	0.075
	Without atypical antipsychotic	145	0.059	0.360	–	–	–	–
Framingham score (LDL)	With atypical antipsychotic	185	0.562	2.335	0.224	(−0.246,	0.693)	0.349
	Without atypical antipsychotic	185	0.286	1.967	–	–	–	–
JALS score	With atypical antipsychotic	147	2.433	8.329	1.442	(−0.435,	3.319)	0.132
	Without atypical antipsychotic	157	1.452	7.427	–	–	–	–

*TG* Triglyceride, *HDL-C* High-density lipoprotein cholesterol, *LDL-C* Low-density lipoprotein cholesterol, *HbA1c* Hemoglobin A1c, *JALS* Japan Arteriosclerosis Longitudinal Study, *SD* Standard deviation, *CI* Confidence interval

Based on multivariate analysis adjusted for the cumulative chlorpromazine equivalent amount and baseline TC or BMI, the changes in TC and BMI were 6.525 mg/dL and 0.380 kg/m^2^ greater, respectively, in the group treated with atypical antipsychotic drugs, and the differences were significant (*p* = 0.037 and 0.011) (Tables 4, 5, 6 and 7). The change in LDL-C was 5.537 mg/dL greater in the group treated with atypical antipsychotic drugs, although there was no significant difference (*p* = 0.058) (Table 8).

There were no significant differences between the two groups in other biomarkers, Framingham score, JALS score, or the onset of diabetes, cardiovascular events, or cardiovascular death (Tables 8 and 9).

**Table 9:** Event rate

Event	Group	N	Mean follow-up (months)	Number of events	HR^a^	95%CI		*P*-value
Diabetes	With atypical antipsychotic	746	8.60	10	1.377	(0.536,	3.539)	0.506
	Without atypical antipsychotic	752	25.38	12	(reference)	–	–	–
CV events	With atypical antipsychotic	889	8.62	12	1.010	(0.455,	2.240)	0.981
	Without atypical antipsychotic	889	24.75	20	(reference)	–	–	–
CV death	With atypical antipsychotic	889	8.70	0	NA^b^	NA	NA	NA
	Without atypical antipsychotic	889	25.36	1	NA	NA	NA	NA

*CV* Cardiovascular; *HR* Hazard ratio, *CI* Confidence interval, *NA* Not applicable

^a^HR was estimated by Cox’s proportional hazards model. Cumulative chlorpromazine equivalent was adjusted as a covariate

^b^HR was unable to be estimated due to the lack of events

### Clinical monitoring

The frequency of monitoring for diabetes and lipid markers in eligible patients (*n* = 3464) was 0.028 times per month (once every 35 to 36 months) and 0.078 times per month (once every 12 to 13 months), respectively, with an average of 0.336 and 0.936 times per patient per annum. There was no significant difference in the frequency of monitoring between treatment groups in the primary endpoint analysis population (*p* = 0.567 and 0.080) (Tables 10 and 11). Seventy-seven and 58% of patients did not have monitoring for diabetes and lipid markers, respectively.

**Table 10:** Frequency of clinical monitoring. Diabetes

Population	Frequency of diabetes monitoring (times/month)^a^			*P*-value
	N	Mean	SD	
Eligible patients	2968^b^	0.028	0.099	–
Primary analysis population (PS matched)				
With atypical antipsychotic	185	0.022	0.064	0.567
Without atypical antipsychotic	185	0.026	0.072	

*PS* Propensity score, *SD* Standard deviation

^a^Eligible patients: total months of examination/total months from index month to the last month available in claims data; Primary analysis population: total months of examination/12 months

^b^Patients with diabetes at index month were excluded

**Table 11:** Frequency of clinical monitoring. Lipid markers

Population	Frequency of lipid marker monitoring (times/month)^a^			*P*-value
	N	Mean	SD	
Eligible patients	3464	0.078	0.184	–
Primary analysis population (PS matched)				
With atypical antipsychotic	185	0.051	0.123	0.080
Without atypical antipsychotic	185	0.076	0.147	

*PS* Propensity score, *SD* Standard deviation

^a^Eligible patients: total months of examination/total months from index month to the last month available in claims data; Primary analysis population: total months of examination/12 months

## Discussion

In this study, we evaluated the difference in cardio-metabolic risk between schizophrenia patients treated with and without atypical antipsychotic drugs using a Japanese employment-based health insurance database. Changes in the Suita score from baseline to 1 year were the primary outcome and were used as an assessment index for cardio-metabolic risk. Our analysis revealed that the changes in the Suita score and risk calculated based on these changes were 0.530 and 0.098% greater, respectively, in the group treated with atypical antipsychotic drugs than in the group treated without atypical antipsychotic drugs, but there was no significant difference (*p* = 0.412 and 0.610). The changes in TC and BMI evaluated as secondary outcomes for cardio-metabolic risk were 6.525 mg/dL and 0.380 kg/m^2^ greater, respectively, in patients treated with atypical antipsychotic drugs, and the differences were significant (*p* = 0.037 and 0.011). Other outcomes did not show any significant differences between the two groups.

Calculation of Suita scores requires information on laboratory values and lifestyle (smoking). As the JMDC database generally includes only claims data on medical institutions, our inclusion criteria inevitably specified that there should have information of medical examinations for patients to be subjected to analysis, and this requirement raises concerns about the external validity of the analysis population. Meanwhile, it is thought that the execution bias could be reduced because those information of medical examinations were not ordered actively deemed to be necessary from the medical institutions. The analysis population in the present study did not differ markedly from those in previous Japanese clinical studies in age, HbA1c, systolic blood pressure, TC, or BMI, with no notable heterogeneity compared with the populations of schizophrenia patients in hitherto reported clinical studies [19–24]. The proportion of male patients was slightly higher in our analysis population. However, subgroup analysis by sex revealed no significant difference in changes in the Suita score between patients treated with and without atypical antipsychotic drugs (male patients: *p* = 0.591; female patients: *p* = 0.471).

In this analysis, there was no effect on changes in the Suita score due to the administration of atypical antipsychotic drugs. One reason may be the limited number of patients and the limited evaluation period. However, the validity of the number of patients was unable to be sufficiently assessed as this was the first study to evaluate 1-year changes in the Suita score in Japanese patients.

Regarding the changes in TC and BMI at 1 year, significant increases (+ 6.525 mg/dL and + 0.380 kg/m^2^, respectively) were observed in patients treated with atypical antipsychotic drugs. However, these increases were only within the normal range and it took 1 year for the changes to occur. It is therefore controversial whether they can be considered a clinically significant increase in cardiovascular risk. However, Someya et al. reported that even a slight increase in BMI within the normal range at around age 20 may predispose one to type 2 diabetes in middle age (32 years later) (The risk of type 2 diabetes was 1.77-times higher in persons with a BMI of ≥21 kg/m^2^ and < 22 kg/m^2^, 2.42-times higher in persons with a BMI of ≥22 kg/m^2^ and < 23 kg/m^2^, and 2.53-times higher in persons with a BMI of ≥23 kg/m^2^ than in persons with a BMI of < 21 kg/m^2^) [25]. Accordingly, further risk assessment is warranted, including long-term outcomes such as cardiovascular events and new-onset diabetes.

The medical examination data requiring the utmost attention in evaluating cardio-metabolic risk is LDL-C, and there was no significant difference in changes in LDL-C between the two groups. However, compared with other clinical studies evaluating 1-year changes in LDL-C due to antipsychotic drugs [26–28], the changes in our analysis population were similar or slightly larger than there results (6.130 mg/dL in patients treated with atypical antipsychotic drugs and 0.454 mg/dL in patients treated without atypical antipsychotic drugs). Our results were obtained in a real-world setting not a strict treatment environment, which highlights the importance of medical examination data monitoring in clinical settings. The mean annual frequency of monitoring diabetes and lipid markers in the eligible patients in this study was 0.336 and 0.936 times per patient (ie, once every 3 years and once a year) and the rate of untested patients during the follow-up period was 76.6 and 57.8%, respectively, suggesting that these medical examination data are not satisfactorily monitored in actual clinical settings. Promotion of the periodic monitoring of blood glucose levels, lipid markers, etc. is important to improve antipsychotic drug treatment.

Several limitations in the present study need to be considered. First, the present analysis focused on health insurance claims data that did not include medical record information. Therefore, not all patients for whom an antipsychotic drug was used on an off-label basis were excluded from the analysis, although we precluded such patients as much as possible by defining the exclusion criteria. Second, the frequency of diabetes monitoring was low, which raises the possibility that the presence of diabetes (defined as fasting plasma glucose ≥7.0 mmol/L (126 mg/dl) or use of any glucose-lowering drug) as a variable for the calculation of Suita scores was underestimated. The medical information databases currently available in Japan do not contain sufficient data regarding clinical laboratory values to achieve the purpose of this study, and it is hoped that a nationwide medical information database including these clinical data will be constructed in the future.

## Conclusions

In this study, significant increases in changes in the Suita score at 1 year by atypical antipsychotic drugs use were not observed, but there were significant increases in TC and BMI in patients treated with atypical antipsychotic drugs.

## Supplementary Information

**Additional file 1: Supplementary Table.** Risk factors considered in propensity scoring.

## Data Availability

The datasets generated and/or analyzed during the current study are not publicly available because it was purchased from a commercial provider (JMDC Inc.), but it is available from the corresponding author on reasonable request.

## Abbreviations

BMI: Body mass index
CHD: Coronary heart disease
CI: Confidence interval
eGFR: Estimated glomerular filtration rate
HbA1c: Hemoglobin A1c
HDL-C: High-density lipoprotein cholesterol
IGT: Impaired glucose tolerance
JALS: Japan Arteriosclerosis Longitudinal Study
LDL-C: Low-density lipoprotein cholesterol
PS: Propensity score
QOL: Quality of life
SD: Standard deviation
SMD: Standardized mean difference
TC: Total cholesterol
TG: Triglyceride
