# Supplementary material for: A propensity score matching analysis for cardio metabolic risk of antipsychotics in patients with schizophrenia using Japanese claims data
Source: BMC Psychiatry. 2020 Dec 9;20:584. doi: 10.1186/s12888-020-02987-1 (PMC7724711; doi:10.1186/s12888-020-02987-1)
Supplement: Supplementary file 1 — Additional file 1: Supplementary Table. Risk factors considered in propensity scoring. [file 12888_2020_2987_MOESM1_ESM.docx]

Research Article

Kusumi et al

# A propensity score matching analysis for cardio metabolic risk of antipsychotics in patients with schizophrenia using Japanese claims data

Ichiro Kusumi

Department of Psychiatry, Hokkaido University Graduate School of Medicine

Kita 15, Nishi 7, Kita-ku, Sapporo, Hokkaido, Japan

Email: ikusumi@med.hokudai.ac.jp

Sachie Inoue

CRECON Medical Assessment Inc.

2-12-15, Shibuya, Shibuya-ku, Tokyo, Japan

Email: inoue@crecon.co.jp

Kenji Baba

Sumitomo Dainippon Pharma Co., Ltd.

13-1, Kyobashi 1-Chome, Chuo-ku, Tokyo, Japan

Email: kenji-1-baba@ds-pharma.co.jp

Tadashi Nosaka

Sumitomo Dainippon Pharma Co., Ltd.

13-1, Kyobashi 1-Chome, Chuo-ku, Tokyo, Japan

Email: tadashi-nosaka@ds-pharma.co.jp

Toshihisa Anzai

Department of Cardiovascular Medicine, Hokkaido University Graduate School of Medicine

Kita 15, Nishi 7, Kita-ku, Sapporo, Hokkaido, Japan

Email: anzai@med.hokudai.ac.jp

Corresponding author:

Ichiro Kusumi

Department of Psychiatry, Hokkaido University Graduate School of Medicine

Kita 15 Nishi 7, Kita-ku, Sapporo, Hokkaido, Japan

Tel: +81 11-706-5160

Fax: +81 11-706-5081

Email: ikusumi@med.hokudai.ac.jp

**Supplementary Table.** Risk factors considered in propensity scoring

| Baseline characteristics | |
| --- | --- |
| Age | |
| Sex | |
| Year of schizophrenia diagnosis | |
| Systolic blood pressure | |
| Diastolic blood pressure | |
| LDL-C | |
| HDL-C | |
| eGFR | |
| BMI | |
| Smoking status | |
| Diabetes | |
| Prescription within a month before baseline | |
| Risk factors for glycometabolism abnormality | Hyperalimentaion |
|  | Corticosteroid |
|  | Interferon preparation |
|  | Thiazide and other diuretics |
|  | Beta-blockers |
|  | Immunosuppressive agents |
|  | Gatifloxacin |
|  | Pentamidine |
|  | Phenytoin |
|  | Antidepressants |
|  | Lipid-lowering agents |
| Risk factors for cardiovascular events (other than ) | Anti-arrhythmia drugs |
|  | Angiotensin converting-enzyme inhibitors |
|  | Angiotensin receptor blockers |
|  | Anticoagulants |
|  | Anti-diabetes drugs |
|  | Aspirin |
|  | Non-aspirin anti-platelet agents |
|  | Calcium-channel blockers |
|  | Digoxin and other inotropic agents |
|  | Loop diuretics |
|  | Nitrates |
|  | Other anti-hypertensive drugs |
|  | Pentoxifylline /related drugs |
| Diagnosis within 6 months before baseline | |
| Risk factors for glycometabolism abnormality | Hypertension |
|  | Lipid metabolism abnormality |
|  | Hyperthyroidism |
|  | Cushing's syndrome |
|  | Chronic hepatitis |
|  | Liver cirrhosis |
|  | Chronic pancreatitis |
|  | Primary aldosteronism |
|  | acromegaly |
|  | Pancreatic cancer |
|  | Liver cancer |
|  | Somatostatinoma |
|  | Glucagonoma |
|  | Pheochromocytoma |
|  | Hemochromatosis |
| Risk factors for cardiovascular events (other than ) | Revascularization |
|  | Myocardial infarction or other coronary heart disease |
|  | Heart failure |
|  | Conduction disorder of arrhythmia |
|  | Valve disorders |
|  | Cerebrovascular disease |
|  | Peripheral vascular disease |
|  | Renal failure |
|  | Chronic obstructive pulmonary disease |
|  | Obesity |
| Associated factors for antipsychotic selection | Other cancer |

**Abbreviations**: LDL-C, low-density lipoprotein cholesterol; HDL-C, high-density lipoprotein cholesterol; eGFR, estimated glomerular filtration rate; BMI, body mass index.
